# Hepatitis Viruses Infection Up-Regulating Fibrosis Signals in Hepatocellular Carcinoma

**DOI:** 10.3390/biomedicines14081717

**Published:** 2026-07-30

**Authors:** Wei-Luen Yen, Yi-Lin Chiu, Hsin-Chung Lin, Hsuan-Wei Chen

**Affiliations:** 1 Division of Rheumatology, Immunology, and Allergy, Department of Internal Medicine, Tri-Service General Hospital, National Defense Medical University, Taipei 11490, Taiwan; 2 Department of Biochemistry, National Defense Medical University, Taipei 11490, Taiwan; 3 Division of Clinical Pathology, Department of Pathology, Tri-Service General Hospital, National Defense Medical University, Taipei 11490, Taiwan; 4 Division of Gastroenterology & Hepatology, Department of Internal Medicine, Tri-Service General Hospital, National Defense Medical University, Taipei 11490, Taiwan

**Keywords:** cryptogenic hepatocellular carcinoma, hepatitis B virus, hepatitis C virus, cirrhosis, fibrosis

## Abstract

**Background and Objectives:** In Taiwan, hepatitis B virus (HBV) or hepatitis C virus (HCV) infections are the most common causes of hepatocellular carcinoma (HCC). However, an increasing number of non-HBV and non-HCV (NBNC) patients developing HCC has been noted in recent years. The pathogenic connection between NBNC status and HCC development remains largely elusive. This study aims to explore the differences in clinical manifestations and pathological findings among HCC patients with HBV, HCV, and NBNC etiologies. **Methods:** This retrospective study analyzed 521 HCC patients with complete hepatic viral profiles at a single medical center in Taiwan between 2016 and 2020. Differentially expressed gene (DEG) analysis, xCell stromal cell estimation, and Gene Set Enrichment Analysis (GSEA) were employed to explore the distinct oncogenic mechanisms between the viral and NBNC groups. **Results:** The final cohort included 38 NBNC-HCC patients. Clinically, the NBNC-HCC patients exhibited a significantly lower FIB-4 index than the HCV-HCC patients (3.191 vs. 6.077, *p* = 0.0019). Furthermore, fewer NBNC-HCC patients presented with imaging-defined cirrhosis compared to HBV-HCC patients (44.4% vs. 68.1%, *p* = 0.0057), and a lower proportion had high Ishak scores (5–6) compared to HCV-HCC patients (31.3% vs. 75.6%, *p* = 0.0025). DEG analysis revealed differential expression in oncogenes (*OIT3*, *AKR1B10*) and liver fibrosis-related genes (*COLEC10*, *CXCL14*, and *LINC01093*). Subsequent GSEA and stromal cell analyses highlighted significant differential enrichment in hepatic stellate cells and vascular endothelial cells. **Conclusions:** NBNC-HCC patients present with significantly lower rates of cirrhosis and fibrosis compared to their viral counterparts. The distinct gene expression profiles and cell-type enrichment features observed between the viral and NBNC groups indicate a divergent, etiology-specific pathogenesis driving NBNC-HCC.

## 1. Introduction

Hepatocellular carcinoma (HCC) is one of the most prevalent malignancies and ranks as the fourth leading cause of cancer-related mortality worldwide [1]. Common risk factors for HCC include viral hepatitis infections, alcohol abuse, and exposure to hepatotoxins. Specifically, hepatitis B virus (HBV) carriers have a 5- to 15-fold increased risk of developing HCC, whereas hepatitis C virus (HCV) carriers face a 17-fold increased risk compared to the general population [2]. In contrast, a distinct subset of HCC patients presents without any known viral or conventional chronic liver disease etiologies; this presentation is clinically referred to as cryptogenic HCC [3].

The majority of HCC cases develop in the context of underlying liver cirrhosis. Among patients with compensated cirrhosis, the annual incidence rate of HCC ranges from 1.4% to 3.3% [4,5,6]. The primary etiologies of cirrhosis in these patients include HBV and HCV infections, alcoholic liver disease, and metabolic dysfunction-associated steatotic liver disease (MASLD, formerly known as NAFLD). Recent epidemiological insights highlight that MASLD has emerged as a leading global cause of chronic liver disease. Furthermore, the coexistence and complex pathophysiological interplay of metabolic dysfunction with or without viral hepatitis have presented a growing challenge, significantly altering the trajectory of liver fibrosis and HCC development [7]. Conversely, approximately 12% to 15% of HCC cases emerge in non-cirrhotic livers. In this non-cirrhotic subset, MAFLD represents the most prevalent underlying liver pathology [8,9].

Following the implementation of the National Antiviral Treatment Program for HBV and HCV in Taiwan, the incidence and mortality rates of hepatocellular carcinoma (HCC) have significantly declined [10]. By contrast, patients with cryptogenic HCC are typically diagnosed at an advanced stage and present with a poor clinical prognosis; thus, early diagnosis for this population is crucial [11]. Previous studies have demonstrated that non-HBV and non-HCV (NBNC) patients who develop HCC—strictly defined in our study as negative for HBsAg, Anti-HBc, and Anti-HCV to effectively exclude latent HBV infections—exhibit a higher prevalence of metabolic syndromes, such as type 2 diabetes mellitus, hypertension, and hyperlipidemia. Furthermore, HCC frequently develops in NBNC patients without underlying liver cirrhosis [12]. It is well-established that the progression of HBV- and HCV-associated HCC is typically accompanied by severe cirrhosis and fibrosis. However, our clinical observations indicate that NBNC-HCC patients present with significantly less cirrhosis and fibrosis. Therefore, we hypothesize that fibrosis signaling plays a pivotal and differential role in the oncogenesis among these three groups, although the precise pathogenic connection between NBNC status and HCC development remains largely elusive.

In this single-center retrospective study, we aim to explore the differences in clinical manifestations, pathological findings in HCC patients with HBV, HCV or NBNC, in which active or latent HBV and HCV were excluded. Furthermore, to investigate our hypothesis regarding the distinct fibrotic tumor microenvironment, we performed Differential Expression Gene analysis, xCell stromal cell analysis, and Gene Set Enrichment Analysis (GSEA). These approaches were utilized to elucidate the underlying etiologies and molecular mechanisms driving HCC development in the NBNC cohort.

## 2. Materials and Methods

### 2.1. Subjects

Patients diagnosed with HCC between January 2016 and December 2020 were enrolled in this study. Diagnoses were confirmed through imaging modalities or histopathological evidence. Clinical data were retrospectively extracted from the cancer registry database maintained by the Division of Cancer Prevention at Tri-Service General Hospital. The recorded parameters included age, sex, body mass index (BMI), lifestyle factors, cancer stage, total bilirubin, international normalized ratio (INR), hepatitis B surface antigen (HBsAg), hepatitis B surface antibody (Anti-HBs), hepatitis C antibody (Anti-HCV), alpha-fetoprotein (AFP) levels, Child–Pugh scores, and Ishak scores. Patients with incomplete medical records were excluded from the analysis.

### 2.2. Study Design

This was a single-center retrospective cohort study. Comprehensive medical histories, including age, sex, BMI, and biochemical data, were systematically reviewed. All patients underwent multidisciplinary evaluations by hepatologists, surgeons, and oncologists. To rigorously exclude patients with latent HBV infections, hepatitis B core antibody (Anti-HBc) data were additionally analyzed. Based on these viral hepatitis serological markers, the enrolled patients were stratified into three distinct groups: the NBNC group (negative for HBsAg, Anti-HBc, and Anti-HCV), the HBV group (positive for HBsAg and negative for Anti-HCV), and the HCV group (negative for HBsAg and positive for Anti-HCV). Patients lacking complete serological profiles were excluded. Laboratory parameters, including aspartate aminotransferase (AST), alanine aminotransferase (ALT), total bilirubin, INR, AFP, platelet counts, serum sodium, and creatinine, were meticulously recorded. Platelet counts were utilized to calculate the Fibrosis-4 (FIB-4) index, which serves as a crucial non-invasive surrogate marker to evaluate the severity of liver fibrosis. Additionally, serum sodium and creatinine levels were used to compute the Model for End-Stage Liver Disease (MELD-Na) score to assess the severity of hepatic dysfunction. The Child–Pugh score and the staging of HCC were also separately recorded.

Imaging studies for liver cirrhosis, including computed tomography (CT), magnetic resonance imaging (MRI), and liver sonography, were evaluated by radiologists and hepatologists. Specifically, radiological cirrhosis was defined based on a combination of CT imaging features (e.g., liver surface nodularity, caudate lobe hypertrophy, and splenomegaly) and Fibroscan elasticity values (Liver Stiffness Measurement [LSM] > 12.5 kPa, corresponding to F4 stage).

Histologic findings for Ishak scoring were reported by pathologists. To ensure that the fibrosis scoring accurately reflected the background liver condition without interference from the tumor, Ishak scores were evaluated using adjacent non-tumor tissues obtained along the safe surgical margins. These histological results were subsequently categorized into low-grade fibrosis (Ishak score 0–4) and severe fibrosis or cirrhosis (Ishak score 5–6) for comparative analysis.

In this study, various standardized scoring systems were employed to comprehensively evaluate liver function, fibrosis burden, and tumor characteristics. Non-invasive liver fibrosis and baseline hepatic dysfunction were assessed using the Fibrosis-4 (FIB-4) index (calculated based on age, AST, ALT, and platelet count) and the Model for End-Stage Liver Disease with Sodium (MELD-Na) score, respectively. Clinical cirrhosis severity was graded using the Child–Pugh score (class A, B, or C). For histological evaluation, liver fibrosis was graded using the Ishak scoring system (stages 0–6) for surgical biopsies in our clinical cohort and the METAVIR system (stages F0–F4) for the external GSE62232 dataset. To ensure comparability, Ishak stages 5–6 were considered clinically equivalent to METAVIR F4 (cirrhosis). Regarding tumor progression, patients were classified according to the TNM staging system, while tumor histological differentiation was graded using the Edmondson–Steiner scoring system (grades I–IV).

### 2.3. Statistical Analysis

Continuous variables are presented as the median and interquartile range (IQR). The results for categorical variables are expressed as numbers and percentages. To rigorously address the imbalanced and relatively small sample sizes across the groups (particularly the NBNC cohort), statistical comparisons for continuous variables were performed using the Kruskal–Wallis test. Categorical variables were compared using the Chi-square test or Fisher’s exact test, as appropriate, depending on the data distribution. All clinical statistical analyses were performed using GraphPad Prism 7.0.

To justify the relatively small sample size of the NBNC-HCC cohort (*n* = 38), a post hoc power analysis was conducted. Assuming α = 0.05 and Power = 0.8, the comparison between the NBNC (*n* = 38) and HBV (*n* = 367) or HCV (*n* = 116) groups was adequately powered to detect a minimum detectable effect of Cohen’s d ≅ 0.5, indicating sufficient statistical power to detect moderate-to-large clinical differences despite the imbalanced sample sizes.

### 2.4. Gene Expression and Cell-Type Profiling

The microarray dataset GSE62232 was downloaded from the NCBI Gene Expression Omnibus (GEO) database (http://www.ncbi.nlm.nih.gov/geo/ (accessed on 7 March 2023)). This dataset contains whole-gene expression profiles from 81 human liver tissue samples, which were subsequently categorized into HBV, HCV, and NBNC (without known etiology) groups for comparative analysis.

To elucidate the transcriptomic differences among these groups, differentially expressed gene (DEG) analysis was conducted using R software (version 4.3.2). The Benjamini–Hochberg procedure was applied to adjust the *p*-values to control the false discovery rate (FDR). This step ensures the statistical robustness of the identified biological signatures. Genes were considered differentially expressed if they met the criteria of an absolute log2 fold change (|log2FC|) ≥ 1 and an FDR < 0.05.

To delineate cellular heterogeneity within the hepatic microenvironment, xCell signatures were utilized [13]. xCell is a quantitative cell-type enrichment analysis based on gene expression data that allows for the computational deconvolution of complex tissues into their constituent cell types. By leveraging comprehensive cell-type-specific gene signatures, this method estimates the relative abundance of 64 distinct immune and stromal cell populations in mixed tissue samples. This provides a robust, quantitative metric that enables us to digitally dissect the specific immune cell infiltrations and microenvironmental remodeling across different HCC etiologies.

Furthermore, to comprehensively represent the biological processes most affected by different etiologies, Gene Set Enrichment Analysis (GSEA) was performed [14]. GSEA addresses the limitations of single-gene testing by analyzing the entire ranked list of genes without pre-filtering, ensuring that the analysis considers subtle expression changes across all genes. This comprehensive approach offers a more inclusive and unbiased view of the systemic gene expression landscape. In this study, GSEA was employed with the Descartes Cell Types and Tissue 2021 database to robustly identify statistically over-represented cell types and states.

## 3. Results

**Figure 1:**
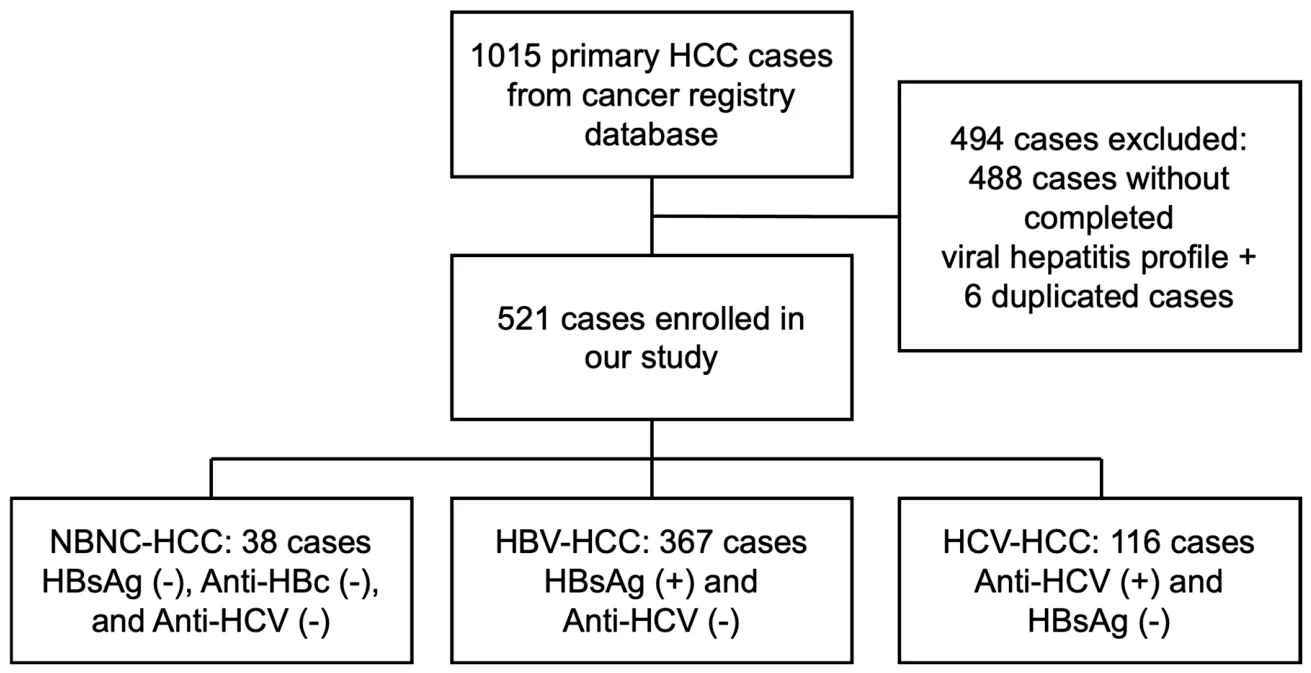
Study flowchart. HCC: Hepatocellular carcinoma; NBNC: Non-HBV and Non-HCV; HBV: Hepatitis B virus; HCV: Hepatitis C virus.

Between 2016 and 2020, a total of 1015 primary HCC patients were initially enrolled. Because our study strictly defined the NBNC cohort as negative for HBsAg, Anti-HBc, and Anti-HCV to effectively exclude patients with latent HBV infections, 488 patients lacking complete serological testing data (particularly Anti-HBc) were excluded from the analysis. Six duplicated cases were also excluded. Consequently, the final analyzed cohort comprised 521 patients. Based on their viral hepatitis serological markers, these patients were categorized into three groups: 367 patients with HBV-HCC (70.44%), 116 patients with HCV-HCC (22.26%), and 38 patients with NBNC-HCC (7.29%) (Figure 1).

### 3.1. Baseline Clinical Characteristics Reveal a Lower Fibrosis Burden in NBNC-HCC Patients

The baseline characteristics of the 521 HCC patients are summarized in Table 1. The NBNC-HCC group (*n* = 38) comprised 24 males and 14 females, with a median age of 64.50 years and a median BMI of 25.01 kg/m^2^. We identified 19 cases of pure metabolic dysfunction-associated steatotic liver disease/metabolic dysfunction-associated steatohepatitis (MASLD/MASH)-associated HCC and 8 cases of overlapping MASLD/MASH and alcohol-associated HCC. The remaining cases consisted of 1 patient with autoimmune liver disease and 10 patients with cryptogenic HCC of unidentified etiology. Regarding baseline clinical assessments, the cohort demonstrated a median total bilirubin of 0.70 mg/dL, a median platelet count of 201.5 × 10^3^/μL, a median FIB-4 index of 2.23, and a median MELD-Na score of 10.80. Liver function was generally well-preserved, with Child–Pugh scores predominantly categorized as grade A (*n* = 20), followed by grade B (*n* = 8) and C (*n* = 1). Finally, the distribution of HCC TNM stages included stage I (*n* = 14), II (*n* = 6), III (*n* = 8), and IV (*n* = 8).

In the HBV-HCC group (*n* = 367), there were 279 males and 88 females. The median age was 63.00 years, and 24.80% had a history of alcohol consumption. The median BMI was 24.03 kg/m^2^. The median total bilirubin was 1.00 mg/dL, the median platelet count was 181.0 × 10^3^/μL, the median FIB-4 index was 2.86, and the median MELD-Na score was 9.65. The Child–Pugh scores were as follows: grade A (*n* = 226), B (*n* = 61), and C (*n* = 26). The TNM stages were stage I (*n* = 138), II (*n* = 40), III (*n* = 131), and IV (*n* = 56).

In the HCV-HCC group (*n* = 116), there were 67 males and 49 females. The median age was 68.00 years, and 20.69% had a history of alcohol consumption. The median BMI was 23.63 kg/m^2^. The median total bilirubin was 0.90 mg/dL, the median platelet count was 145.5 × 10^3^/μL, the median FIB-4 index was 3.97, and the median MELD-Na score was 9.49. The Child–Pugh scores were grade A (*n* = 62), B (*n* = 22), and C (*n* = 7). The TNM stages were stage I (*n* = 44), II (*n* = 28), III (*n* = 30), and IV (*n* = 13).

Patients with NBNC-HCC exhibited a significantly lower FIB-4 index compared to the HCV-HCC group (2.23 vs. 3.97; *p* = 0.0012) and significantly higher platelet levels (201.5 vs. 145.5 × 10^3^/μL; *p* = 0.0192). Additionally, the NBNC-HCC group had a significantly lower total bilirubin level compared to the HBV-HCC group (0.70 vs. 1.00 mg/dL; *p* = 0.0486). Furthermore, there were no statistically significant differences in MELD-Na scores across the NBNC, HBV, and HCV groups (*p* > 0.9999), indicating comparable baseline hepatic dysfunction among the cohorts.

### 3.2. Radiological and Histopathological Assessments Confirm Distinct Fibrotic Phenotypes Across Etiologies

**Figure 2:**
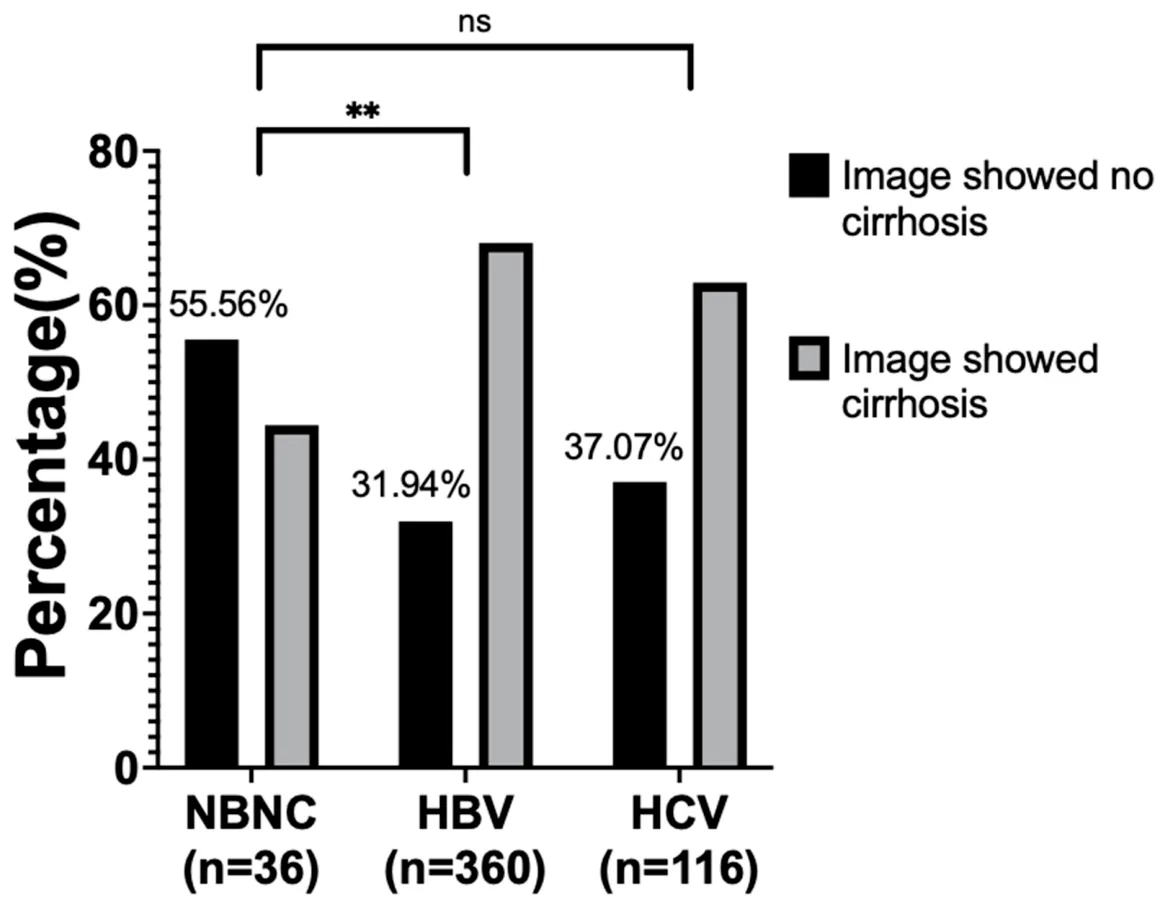
Comparison of percentage of image showed no cirrhosis between the NBNC (*n* = 36), HBV (*n* = 360), and HCV (*n* = 116) groups. ** *p* = 0.0057. NBNC: Non-HBV and Non-HCV; HBV: Hepatitis B virus; HCV: Hepatitis C virus.

Regarding the evaluation of liver cirrhosis, a total of 36 NBNC-HCC, 360 HBV-HCC, and 116 HCV-HCC patients underwent imaging studies. The results demonstrated that 55.56% of the NBNC-HCC patients exhibited no radiological evidence of cirrhosis, a proportion notably higher than the 31.94% observed in the HBV-HCC group and 37.07% in the HCV-HCC group. There was a statistically significant difference in the proportion of non-cirrhotic patients between the NBNC-HCC and HBV-HCC groups (*p* = 0.0057) (Figure 2).

**Figure 3:**
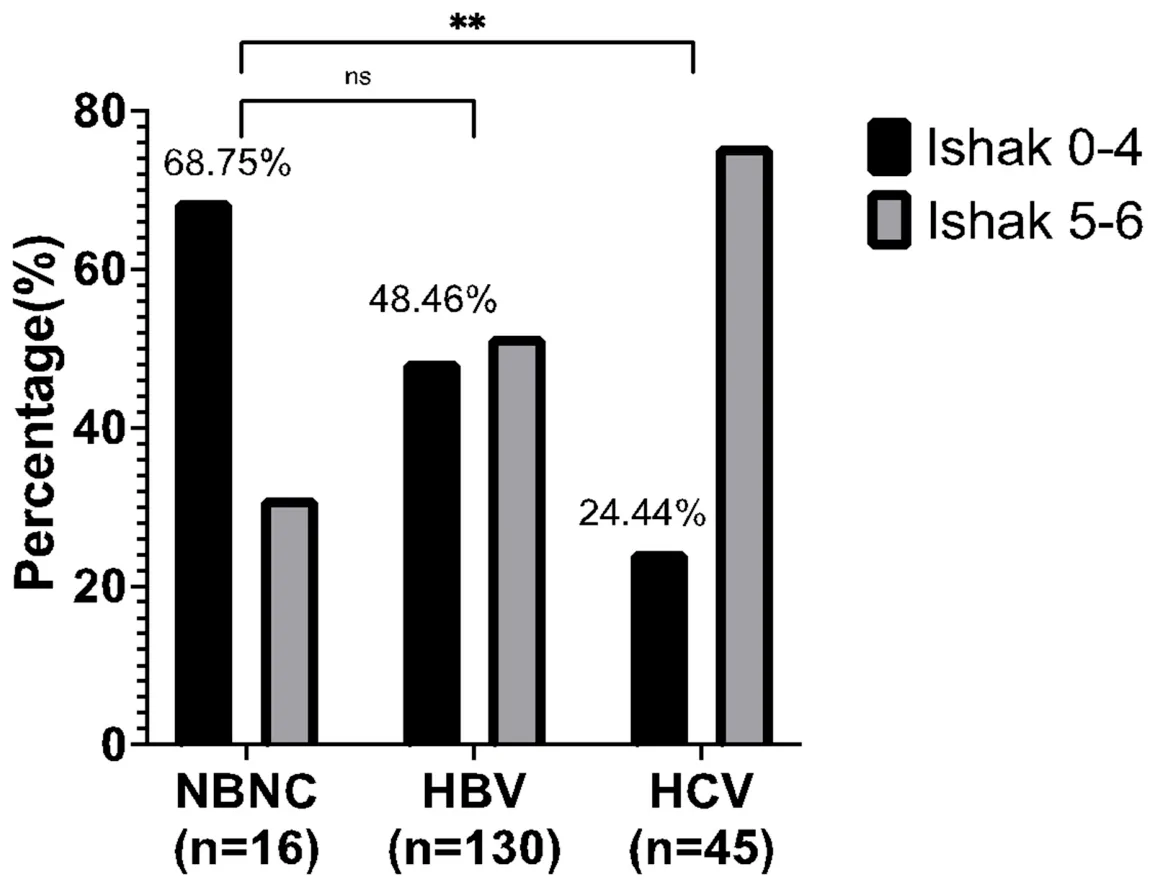
Comparison of percentage of Ishak score 0–4 versus Ishak score 5–6 between the NBNC (*n* = 16), HBV (*n* = 130), and HCV (*n* = 45) groups. ** *p* = 0.0025. NBNC: Non-HBV and Non-HCV; HBV: Hepatitis B virus; HCV: Hepatitis C virus.

For histological assessment, 16 NBNC-HCC, 130 HBV-HCC, and 45 HCV-HCC patients had available Ishak scores. To accurately reflect the background liver condition, these scores were evaluated using adjacent non-tumor tissues obtained along safe surgical margins. In the NBNC-HCC cohort, 68.75% of the patients presented with low-grade fibrosis (Ishak scores 0–4). In contrast, these proportions were 48.46% in the HBV-HCC group and only 24.44% in the HCV-HCC group. The higher proportion of low-grade fibrosis in the NBNC-HCC cohort was statistically significant when compared to the HCV-HCC group (*p* = 0.0025) (Figure 3).

### 3.3. Transcriptomic Profiling Demonstrates Divergent DEG Signatures Between Viral and NBNC Cohorts

**Figure 4:**
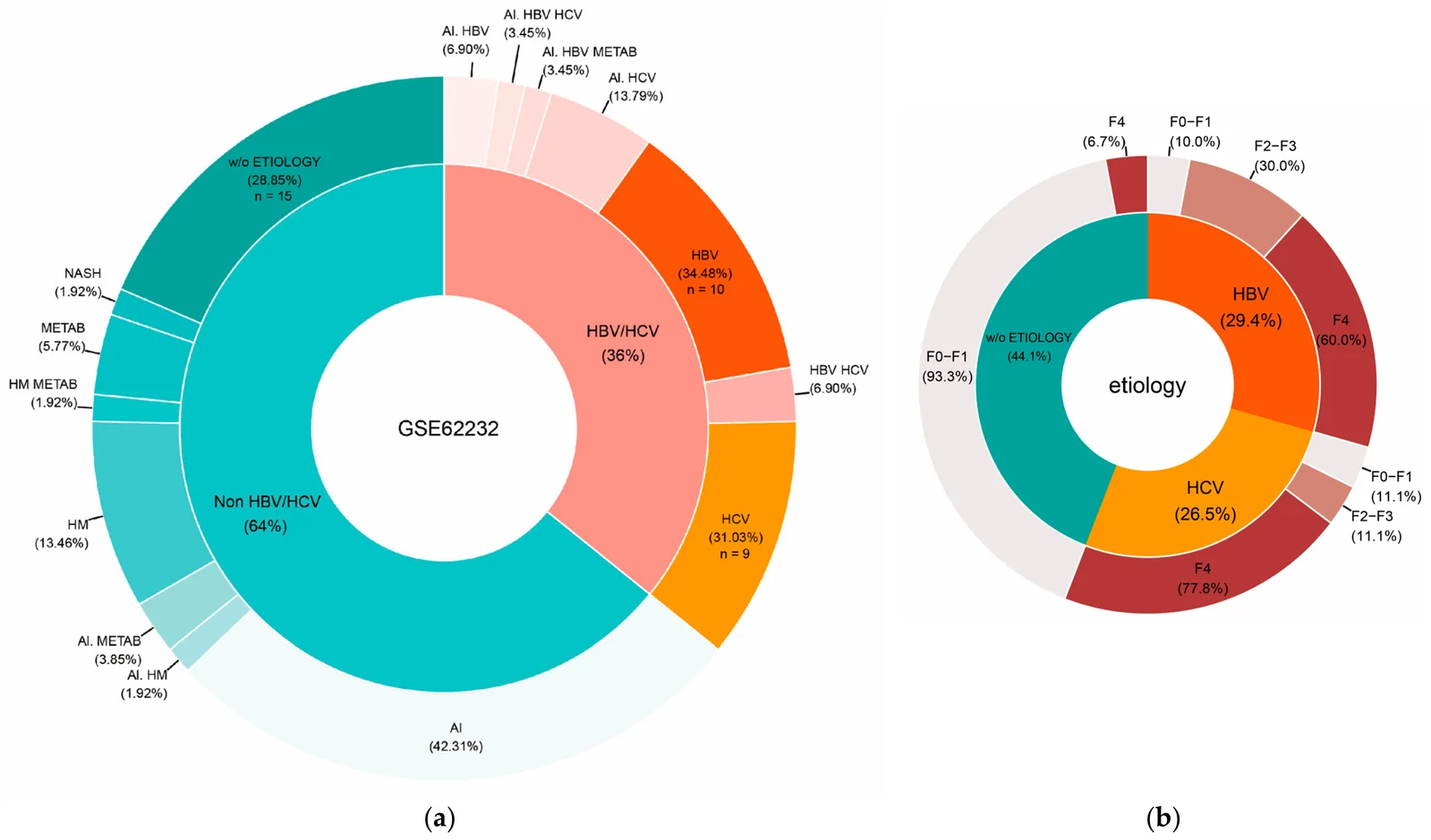
The multilevel donut charts present proportion of HCC etiologies in database GSE62232 and exclude alcoholism, metabolic syndrome, hepatic metastasis, non-alcoholic steatohepatitis, and combined HBV and HCV groups. (**a**) In database GSE62232, a total of 81 patients, 10 HBV patients (12.35%), 9 HCV patients (11.11%), and 15 HCC patients without known etiology (w/o etiology, 18.52%) were highlighted. (**b**) In HBV, HCV, and w/o etiology groups, METAVIR score is presented in the outer ring. Proportion of F4 in HBV (60%), HCV (77.8%) and w/o etiology (6.7%) groups is highlighted. Al: Alcoholism; METAB: Metabolic syndrome; HM: Hepatic metastasis; NASH: Non-alcoholic steatohepatitis; HBV: Hepatitis B virus; HCV: Hepatitis C virus; HCC: Hepatocellular carcinoma; w/o: Without.

From the GSE62232 dataset, we analyzed 15 HCC patients without a known etiology (w/o etiology), 10 patients with HBV-HCC, and 9 patients with HCV-HCC (Figure 4). To align this cohort with our clinical group definitions, 47 patients with alcoholism, metabolic syndrome, hepatic metastasis, MASH, or combined HBV and HCV infections were excluded. Regarding the METAVIR fibrosis scores, 93.3% of the patients in the w/o etiology group presented with mild-to-moderate fibrosis (F0–F3). In contrast, only 40.0% of the HBV group and 22.2% of the HCV group exhibited F0–F3 scores. The proportion of patients with F0–F3 scores was significantly higher in the w/o etiology group compared to both the HBV and HCV groups (*p* < 0.05). As summarized in Table 2, there were no statistically significant differences in gender or Edmondson scores across the groups (*p* > 0.900); however, the age distribution showed a statistically significant difference (*p* = 0.005).

**Figure 5:**
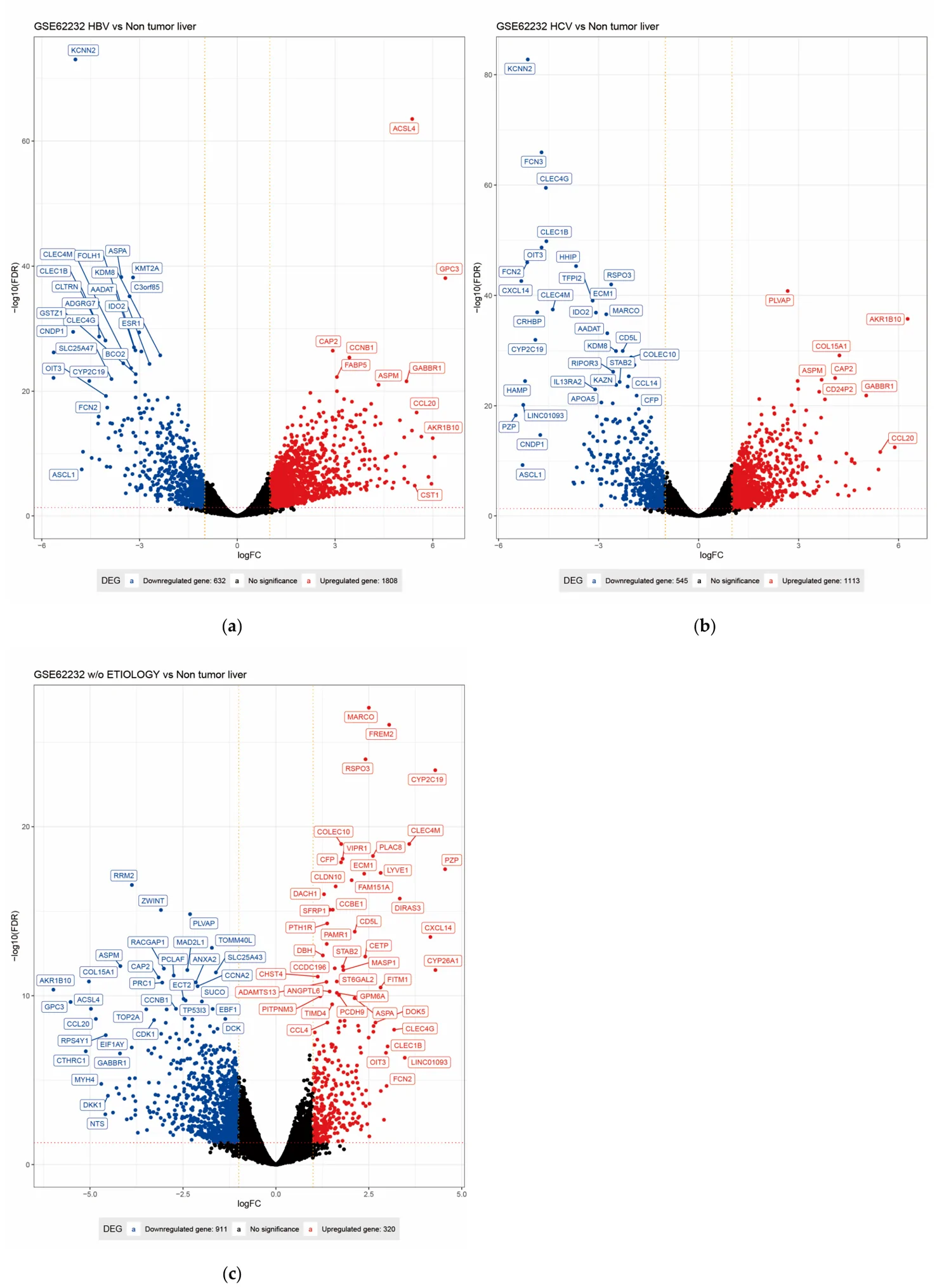
Volcano plot showing differentially expressed genes, with upregulation in red, downregulation in blue, based on false discovery rate (FDR) < 0.05 (as -log10(FDR) in y-axis) and the log2 fold change > 1 (x-axis). (HBV, *n* = 10; HCV, *n* = 9; w/o etiology, *n* = 15). (**a**) HBV vs. non-tumor group showed 1808 upregulated genes and 632 downregulated genes. (**b**) HCV vs. non-tumor group showed 1113 upregulated genes and 545 downregulated genes. (**c**) W/o etiology vs. non-tumor group showed 320 upregulated genes and 911 downregulated genes. Significant genes are highlighted. HBV: Hepatitis B virus; HCV: Hepatitis C virus.

To evaluate the differentially expressed genes (DEGs), volcano plots were generated to compare these three specific groups against non-tumor tissues (Figure 5). The plots represent the −log10-transformed adjusted *p*-values (false discovery rate, FDR) on the y-axis against the log2 fold change on the x-axis. In the w/o etiology group, 320 genes were upregulated, and 911 genes were downregulated. In the HBV group, 1808 genes were upregulated, and 632 genes were downregulated. In the HCV group, 1113 genes were upregulated, and 545 genes were downregulated. Notably, specific genes such as *CLEC1B*, *CLEC4G*, *CLEC4M*, *CYP2C19*, *FCN2*, *LINC01093*, and *OIT3* were significantly upregulated in the w/o etiology group but downregulated in both the HBV and HCV groups. Conversely, *AKR1B10*, *ASPM*, *CAP2*, *CCL20*, and *GABBR1* were downregulated in the w/o etiology group but upregulated in the viral cohorts.

### 3.4. Cell-Type Enrichment Analysis Highlights Etiology-Specific Immune Responses and Robust Fibrotic Remodeling in NBNC-HCC

**Figure 6:**
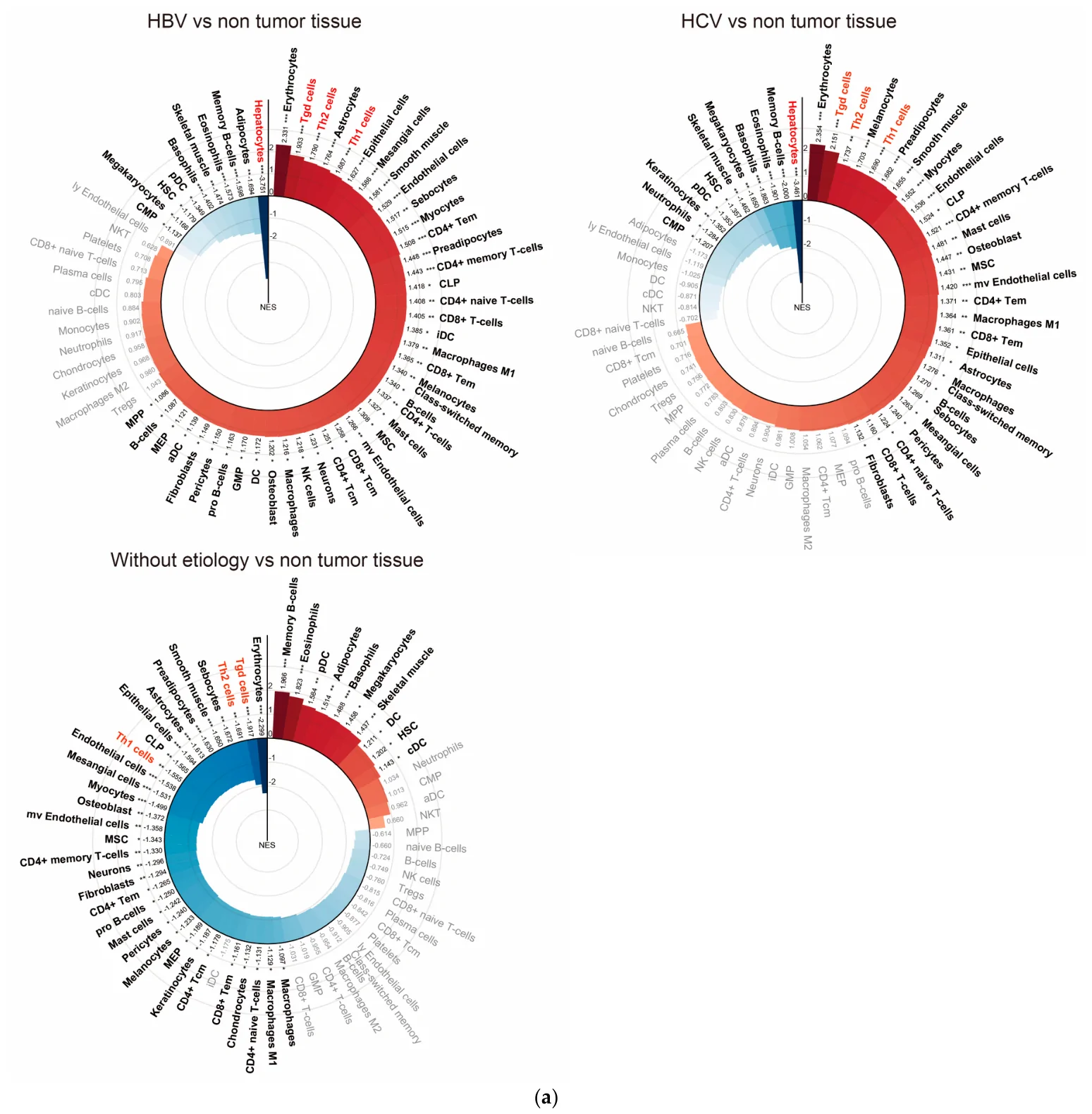

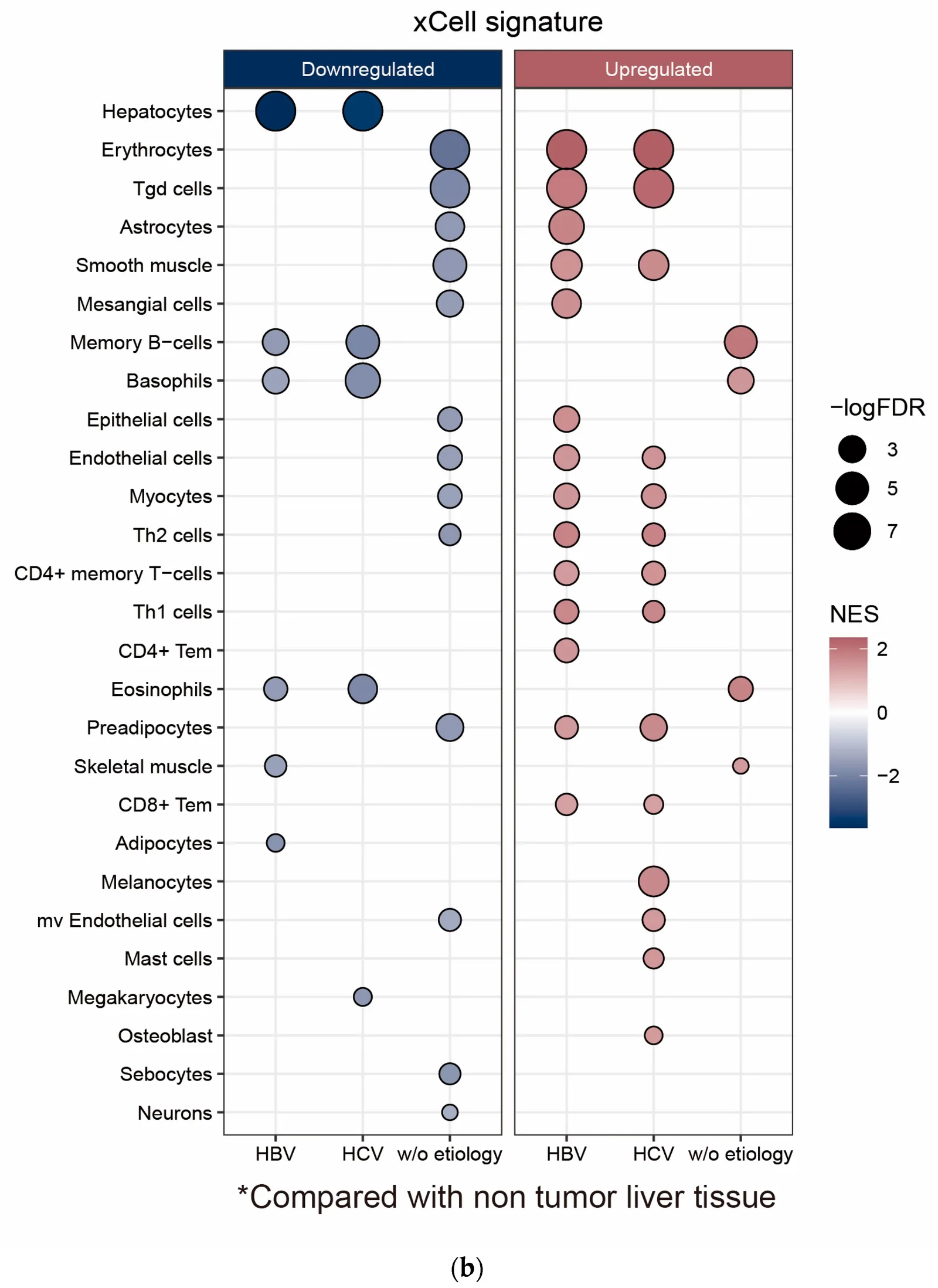
Liver and immune cellular profiles enrichments in xCell signature (HBV, *n* = 10; HCV, *n* = 9; w/o etiology, *n* = 15). (**a**) Radial plot of HBV, HCV, and w/o etiology groups versus non-tumor tissue from xCell with red bars for positive and blue for negative enrichment. Gene sets with FDR < 0.25 are in bold, with significance levels: *, **, and *** for FDR < 0.25, <0.05, and <0.01, respectively. (**b**) Dot plot highlighted 27 significant cellular enrichment of HBV, HCV and w/o etiology group in xCell signature; The size of the dots represents the −logFDR, indicating the significance of the cellular enrichment. The color of the dots, represented using the red–white–blue color scale, indicates the normalized enrichment score in each cell type, with red indicating a higher score. Tgd cell: Gamma delta T cell; Th: Helper T; DC: Dendritic cells; NK cells: Natural killer cells; CD: Cluster of differentiation; CLP: Common lymphoid progenitors; HSC: Hepatic stellate cells; MSC: Mesenchymal stem cells; MEP: Megakaryocyte–erythroid progenitor cells; MPP: Multipotent progenitors; CMP: Common myeloid progenitors; GMP: Granulocyte myeloid progenitors.

Specifically, utilizing the xCell signature database, we observed that the normalized enrichment scores (NES) and false discovery rates (FDR) for various immune and stromal cells exhibited similar trends in the HBV and HCV cohorts, whereas distinct profiles were observed in the group without known etiology (w/o etiology) (Figure 6). In the HBV and HCV groups, hepatocytes—the primary parenchymal cells of the liver—showed consistently negative NES, indicating significant depletion due to severe chronic injury (HBV: NES = −3.64, FDR < 0.001; HCV: NES = −3.36, FDR < 0.001). Conversely, erythrocytes and gamma delta T (Tgd) cells were significantly over-represented in both viral groups (HBV Tgd: NES = 1.91, FDR < 0.001; HCV Tgd: NES = 2.15, FDR < 0.001). Furthermore, memory B-cells and basophils, which are critical components of the adaptive and innate immune responses, were significantly downregulated in both viral cohorts (HBV memory B-cells: NES = −1.62, FDR = 0.0011; HCV memory B-cells: NES = −1.94, FDR < 0.001), suggesting a compromised humoral immune response under persistent viral infection. In contrast, the w/o etiology group displayed a different immunological landscape. Hepatocytes did not show a significant negative enrichment, implying a relative preservation of parenchymal cells without severe cirrhotic progression. Additionally, erythrocytes and Tgd cells were under-represented (erythrocytes: NES = −2.31, FDR < 0.001; Tgd: NES = −1.91, FDR < 0.001), while memory B-cells (NES = 1.95, FDR < 0.001) and basophils (NES = 1.49, FDR = 0.0011) were significantly over-represented, reflecting a distinct immune state likely driven by metabolic inflammation rather than viral exhaustion.

**Figure 7:**
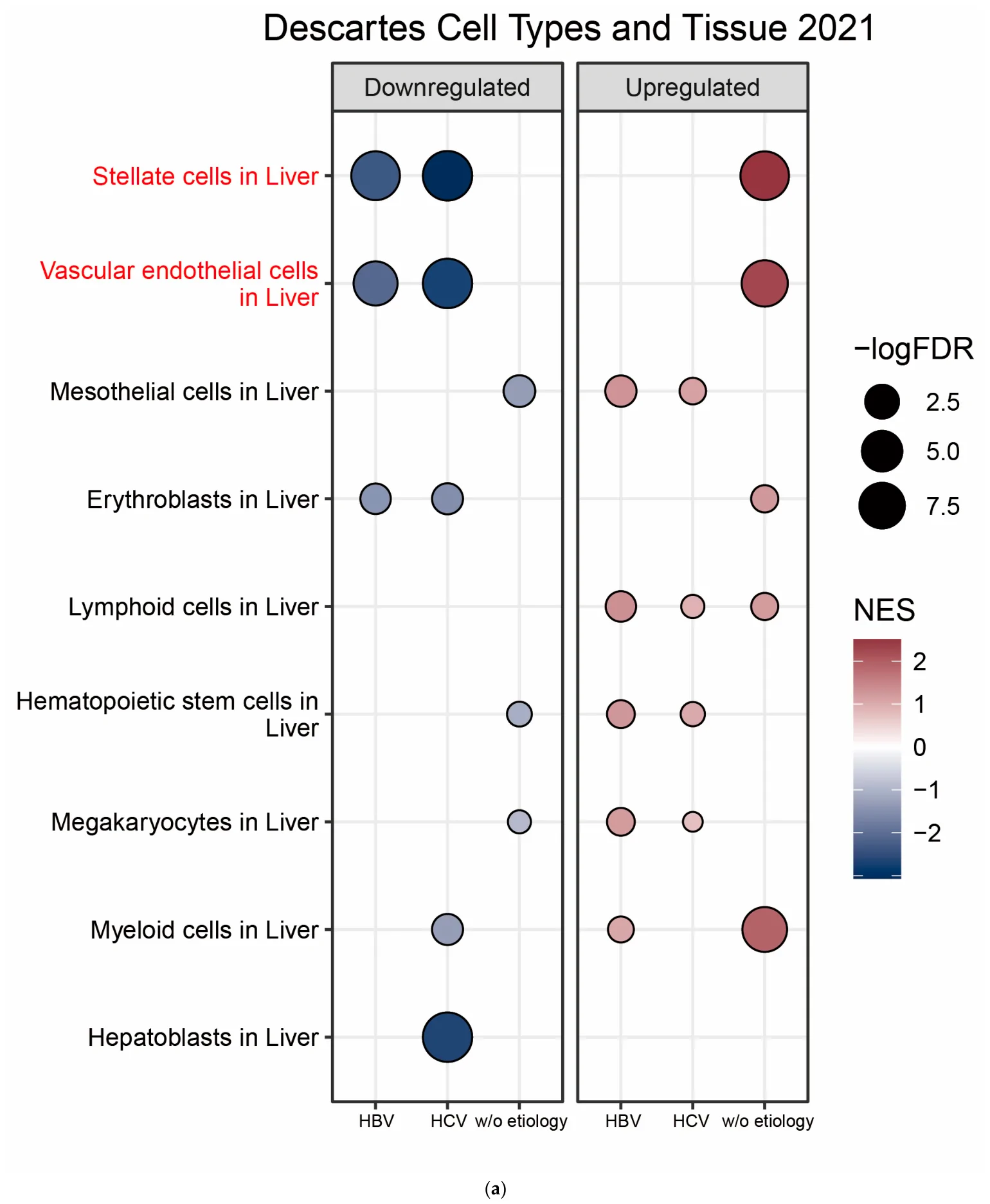

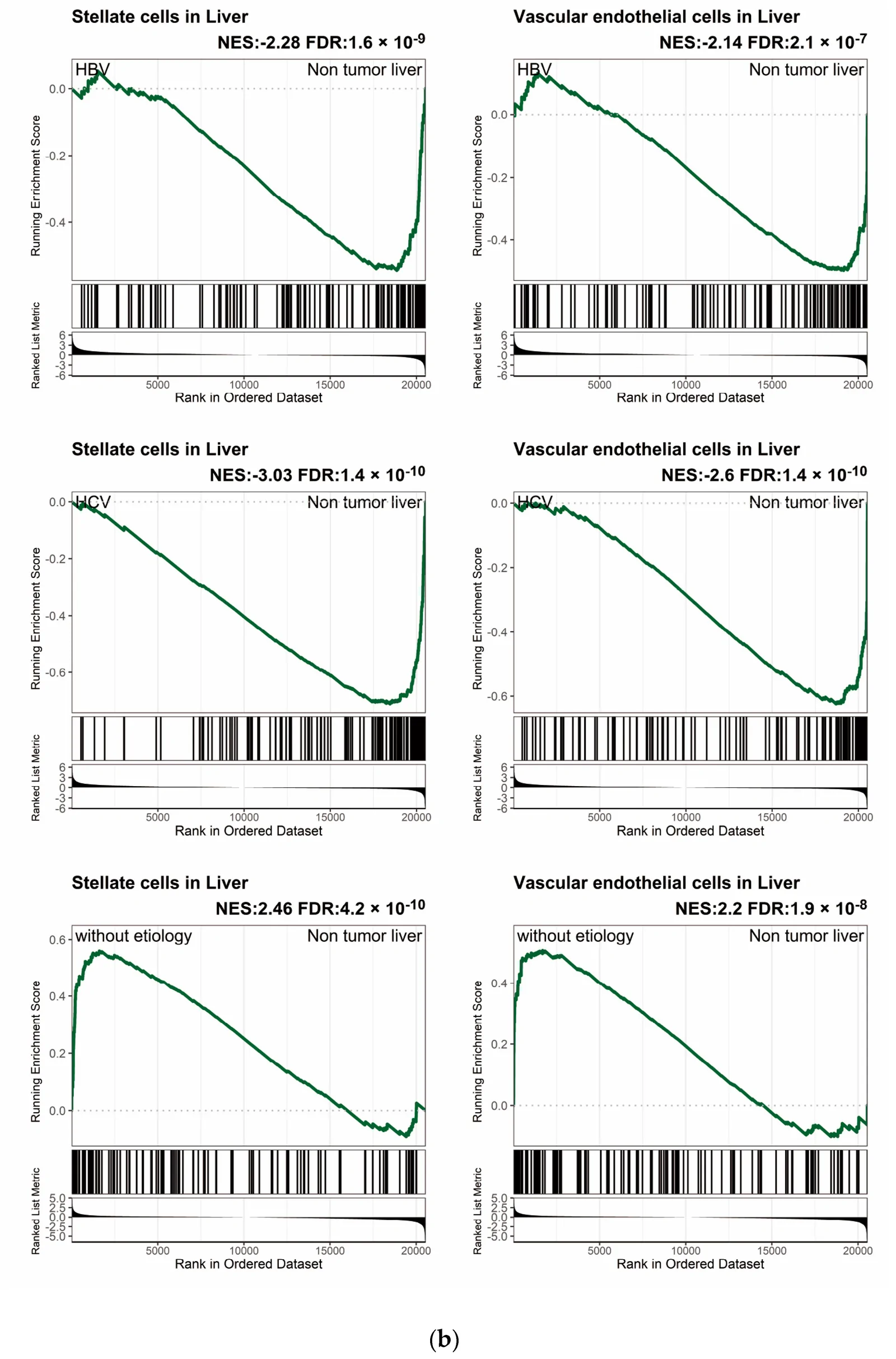
Liver and immune cellular profiles enrichments in Descartes Cell Types and Tissue 2021 (HBV, *n* = 10; HCV, *n* = 9; w/o etiology, *n* = 15). (**a**) Dot plot representing the association with different cells in liver, sourced from the Descartes Cell Types and Tissue 2021 database. The size of the dots represents the -logFDR, indicating the significance of the cellular enrichment. The color of the dots, represented using the red–white–blue color scale, indicates the NES in each cell type, with red indicating a higher score. (**b**) The Gene Set Enrichment Analysis plots present top 2 cell types by NES contrasting HBV, HCV and w/o etiology groups (left) with non-tumor liver group (right). FDR: False discovery rate; NES: Normalized enrichment score; HBV: Hepatitis B virus; HCV: Hepatitis C virus.

To further evaluate statistically over-represented cell populations associated with liver cirrhosis, GSEA was performed using the Descartes Cell Types and Tissue 2021 database (Figure 7). Hepatic stellate cells (HSCs) and vascular endothelial cells emerged as the most critically highlighted populations. In the viral cohorts, both cell types were significantly downregulated compared to the non-tumor group (HBV: HSCs NES = −2.28, FDR = 1.6 × 10^−9^; vascular endothelial cells NES = −2.14, FDR = 2.1 × 10^−7^; HCV: HSCs NES = −3.03, FDR = 1.4 × 10^−13^; vascular endothelial cells NES = −2.60, FDR = 1.4 × 10^−13^). By striking contrast, both HSCs (NES = 2.44, FDR = 4.2 × 10^−13^) and vascular endothelial cells (NES = 2.20, FDR = 1.9 × 10^−8^) were robustly upregulated in the w/o etiology group, highlighting a distinct fibrotic and angiogenic microenvironmental remodeling. Additionally, erythroblasts followed this specific pattern, being upregulated in the w/o etiology group but downregulated in the viral cohorts. Conversely, mesothelial cells, hematopoietic stem cells, and megakaryocytes were upregulated in the HBV and HCV groups but downregulated in the w/o etiology group. Lymphoid cells exhibited uniform upregulation across all three etiologies.

## 4. Discussion

In this single-center retrospective study, an initially enrolled cohort of 1015 primary HCC patients was rigorously filtered to a final analyzed cohort of 521 patients, who were subsequently categorized into HBV, HCV, and NBNC-HCC groups based on their viral serology. By integrating detailed clinical, radiological, and histopathological data with multi-dimensional transcriptomic profiling (including DEG, xCell, and GSEA), we quantitatively delineated the distinct pathophysiological mechanisms characterizing the NBNC cohort, effectively addressing a critical knowledge gap regarding non-viral HCC.

Our clinical findings reveal notable differences in the fibrosis burden among the three groups. Patients with NBNC-HCC exhibited a significantly lower median FIB-4 index and higher platelet levels compared to the HCV-related HCC cohort. Notably, the MELD-Na scores showed no statistically significant differences across all groups, indicating comparable baseline hepatic dysfunction without overt decompensation. These differences in fibrosis markers suggest that the underlying fibrotic progression in NBNC-HCC is significantly less severe than in viral etiologies. In contrast, the HCV-related cohort displayed the highest FIB-4 index and lowest platelet counts, reflecting advanced fibrotic processes and aggressive chronic liver injury. The critical need for long-term, non-invasive fibrosis assessment (such as liver stiffness monitoring) in HCV patients has been well-documented, particularly regarding the dynamic improvement of local tissue stiffness following viral clearance [15]. Our data reinforce the severe fibrotic baseline typical of HCV, which sharply contrasts with the NBNC cohort.

Radiological and histopathological evaluations further validated the distinct fibrotic phenotypes across these groups. Imaging studies demonstrated that a significantly higher percentage of NBNC-HCC patients exhibited no clinical cirrhosis compared to the HBV and HCV groups. Similarly, histopathological evaluation using Ishak scoring—specifically assessed on adjacent non-tumor tissues rather than the tumor itself to accurately reflect the background liver condition—revealed that a substantially greater proportion of NBNC-HCC patients presented with low-grade fibrosis. These observations strongly indicate that severe liver cirrhosis and fibrotic progression occur less frequently or evolve much more slowly in NBNC-HCC. Clinically, this distinct, less-fibrotic microenvironment in NBNC-HCC might necessitate tailored surveillance strategies, as traditional screening guidelines heavily rely on cirrhotic progression, potentially delaying early diagnosis in this specific subpopulation.

While the oncogenic mechanisms driving HBV- and HCV-related HCC are relatively well-characterized—such as HCV compartmentalization and viral quasispecies promoting higher tumor proliferation rates [16], or HBV-host genomic interactions serving as predictive markers [17]—the exact pathogenesis of NBNC-HCC remains largely elusive. Our macroscopic clinical and pathological findings confirm that NBNC-HCC diverges significantly from the traditional viral hepatitis-to-cirrhosis trajectory. To elucidate this unique etiology at the molecular level and to identify potential predictive biomarkers for treatment responses, we employed comprehensive transcriptomic profiling, wherein GSEA and cellular enrichment analyses played a pivotal role.

In the GSE62232 cohort comprising 81 HCC patients categorized into HBV, HCV, and w/o etiology groups, the transcriptomic features demonstrated a strong concordance with our clinical findings. Specifically, patients in the w/o etiology group exhibited significantly lower METAVIR fibrosis scores, with a markedly higher proportion of patients in the F0–F3 stages compared to the HBV and HCV groups. Because Ishak stages 5–6 are clinically equivalent to a METAVIR score of F4, this confirms that the background histological fibrosis was also substantially lower in the w/o etiology group. Furthermore, DEG analysis of this database revealed distinct transcriptomic signatures: genes such as *CLEC1B*, *CLEC4G*, *CLEC4M*, *CYP2C19*, *FCN2*, and *OIT3* were upregulated, whereas *AKR1B10*, *ASPM*, *CAP2*, *CCL20*, and *GABBR1* were downregulated in the w/o etiology group. These expression patterns were entirely opposite to those observed in the HBV and HCV cohorts. The differential regulation of oncogenes like *OIT3* [18] and *AKR1B10* [19] across these groups likely represents divergent mechanisms of hepatic oncogenesis. Additionally, liver fibrosis-related genes, including *COLEC10* [20], *CXCL14* [21], and *LINC01093* [22], were upregulated in the w/o etiology group but downregulated in the HCV group. Beyond these isolated gene expressions, evaluating cell-type enrichment profiles provides a more comprehensive understanding of the tumor microenvironment.

Cell-type enrichment analysis using xCell revealed that T-cells (Tgd, Th1, Th2) dominated the viral cohorts, indicating predominantly T-cell-mediated chronic inflammation and immune dysregulation. Conversely, memory B cells and basophils were enriched in the w/o etiology group but depleted in viral cohorts. This depletion reflects a compromised humoral immune response, consistent with previous studies demonstrating that HCV-induced cirrhosis leads to profound memory B-cell loss and dysfunction. [23] In contrast, their significant enrichment in the w/o etiology cohort signifies an immune state driven by metabolic inflammation. This aligns with recent evidence that specific memory and regulatory B cell subsets exert strong immunosuppressive and protumorigenic effects during MASLD-HCC progression. [24] Furthermore, the relative preservation of hepatocytes in the w/o etiology group supports our clinical observation that NBNC-HCC largely bypasses the severe cirrhotic parenchymal depletion typical of viral HCC. [25].

**Figure 8:**
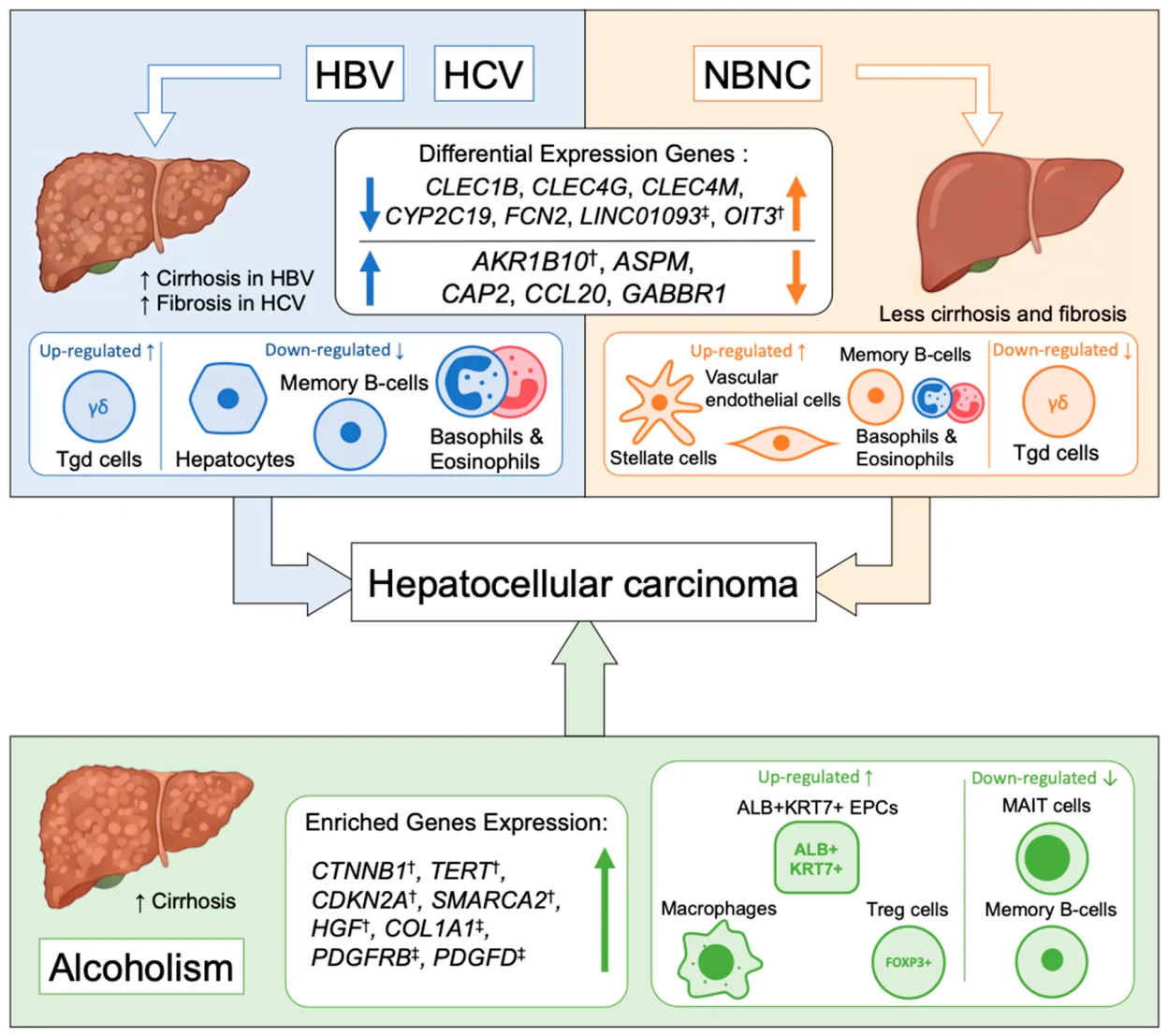
Distinct pathogenetic pathways in HBV-, HCV-, MASH, and ALD patients to develop hepatocellular carcinoma. † Oncogene; ‡ Gene associated with liver fibrosis. HBV: Hepatitis B virus; HCV: Hepatitis C virus; MASH: Metabolic dysfunction-associated steatohepatitis; ALD: Alcohol-associated liver disease; Tgd cell: Gamma-delta T-cell; ALB: Albumin; KRT7: Keratin 7; EPC: Epithelial progenitor cell; Treg: Regulatory T cell; MAIT cell: Mucosal-associated invariant T cell.

Crucially, GSEA mapping demonstrated robust upregulation of HSCs and vascular endothelial cells in the w/o etiology group. Unlike viral HCC, NBNC-HCC is primarily driven by metabolic stress, which robustly induces HSC activation and angiogenesis. This is highly consistent with recent multiomics data showing metabolic stress directly accelerates HSC autocrine signaling and endothelial capillarization without requiring severe global cirrhosis [26]. This etiology-specific adaptation fosters a unique fibrotic and angiogenic tumor microenvironment characterized by high stromal density. In contrast, alcohol-associated liver disease (ALD), another major contributor to the non-viral HCC spectrum, follows a distinct traditional trajectory. Recent exome sequencing and single-cell transcriptomic studies indicate that ALD progression strongly converges on the Wnt/β-catenin oncogenic pathway and classical cancer driver mutations (e.g., CTNNB1, TERT, and CDKN2A), which in turn orchestrate severe cirrhotic remodeling and the specific expansion of ALB + KRT7+ epithelial cells (Figure 8). [27,28] These distinct cellular and molecular profiles underscore the complexity of non-viral liver oncogenesis, offering valuable insights into differential pathogenesis and targeted therapeutic interventions.

This study has several limitations. First, its single-center, retrospective design restricts dynamic clinical interventions and real-time efficacy tracking, while introducing selection and referral biases vulnerable to imaging protocols and surgical candidacy over 2016–2020. Second, the relatively small sample size of the NBNC-HCC cohort (*n* = 38) and the overall clinical heterogeneity may reduce statistical power, calling for caution in data interpretation despite rigorous statistical adjustments. Third, the clinical treatment regimens in our retrospective cohort were highly heterogeneous, which prevented a direct and unbiased comparison of treatment responses and clinical outcomes across the different etiologies. Fourth, etiologic assignment may be imperfect: the NBNC category aggregates biologically distinct conditions (e.g., MASLD/MASH, alcohol-related, autoimmune, cryptogenic), and occult HBV or resolved HCV could lead to misclassification. Fifth, fibrosis characterization mixed surrogate indices (FIB-4, platelets), imaging, and limited histology; not all patients underwent each modality, creating spectrum and verification bias, and Ishak-to-METAVIR conversion compresses staging and may underestimate cirrhosis. Sixth, external validation leveraged GSE62232 and deconvolution (xCell) mapping to an atlas (Descartes), which are sensitive to batch effects and tissue composition differences; the “without etiology” group in GSE62232 may not perfectly recapitulate NBNC-HCC. Finally, the identified DEG signatures and cellular enrichments (e.g., HSCs and vascular endothelial cells) remain correlative. Due to the unavailability of physical clinical tissue samples in this retrospective database study, we were unable to perform independent experimental validation at the protein level—such as quantitative PCR (qPCR), Western blotting, or immunohistochemistry (IHC)—to biologically confirm these molecular findings.

Future work should include prospective, multi-center validation cohorts with larger sample sizes and harmonized data capture. Rigorous viral reservoir testing and granular NBNC subtyping (including MASLD/MASH, alcohol-related, and autoimmune conditions) are essential, alongside standardized fibrosis assessments—such as biopsy combined with elastography using uniform scoring—to minimize verification bias. Crucially, independent experimental validation using approaches like qPCR, Western blotting, and IHC is urgently needed to confirm the biological significance of the identified gene signatures. Parallel protein validation, phospho-signaling, and circulating biomarker assays can bridge tissue signals to noninvasive stratification. Finally, longitudinal analyses must evaluate these unique microenvironmental indicators and cell-state signatures as precise predictive biomarkers for treatment efficacy. Mechanism-guided, etiology-stratified clinical trials can then properly assess targeted therapeutic strategies and treatment responses specifically tailored for the NBNC-HCC subpopulation.

## 5. Conclusions

This single-center cohort delineates distinct clinical, fibrotic, and molecular landscapes across HBV-HCC, HCV-HCC, and NBNC-HCC. Clinically, NBNC-HCC showed a significantly lower fibrosis burden—reflected by lower FIB-4, higher platelets, less imaging-defined cirrhosis, and lower Ishak grades—suggesting a slower fibrotic trajectory and a unique role of fibrosis signaling in its oncogenesis compared with viral cohorts. External transcriptomic validation echoed these differences, with “without etiology” cases exhibiting lower METAVIR stages and a divergent gene program, alongside immune-cell contrasts and greater hepatocyte enrichment. Cell-type-resolved analyses further highlighted etiology- and cell-specific signatures, particularly the robust upregulation of HSCs and vascular endothelial cells, implicating differential axes of regeneration, fibrogenesis, angiogenesis, and immunomodulation. Serving as a crucial first step, these findings refine the heterogeneity of HCC beyond viral status and unravel the complex non-viral tumor microenvironment. Regarding future clinical applications, the identified microenvironmental profiles and candidate biomarkers hold great potential to optimize tailored surveillance patterns, improve early risk stratification, and guide the development of etiology-specific targeted therapies for NBNC disease. Ultimately, these promising mechanism-guided avenues merit prospective, multi-center, and functional experimental validation for successful clinical translation.

## Figures and Tables

**Table 1:** Comparison of the characteristics Between NBNC, HBV, and HCV and by age, sex, BMI, alcohol, creatinine, total bilirubin, INR AFP, ALT, PLT, Fib-4 index, MELD-Na score, Child–Pugh score, and HCC stage.

	NBNC, *n* = 38	HBV, *n* = 367	HCV, *n* = 116	*p* Value	
				NBNC versus HBV	NBNC versus HCV
Age, years (median, IQR)	64.50 (54.75–72.25)	63.00 (55.00–71.00)	68.00 (60.00–76.00)	>0.9999	0.2149
Sex, cases (Male/Female)	24/14	279/88	67/49	0.1141	0.5756
BMI, kg/m^2^ (median, IQR)	25.01 (21.75–27.30)	24.03 (21.29–26.55)	23.63 (21.81–25.80)	>0.9999	0.7309
Alcohol, cases (Alcohol/Non-alcohol)	8/30	91/276	24/92	0.6954	>0.9999
Underlying etiologies of NBNC, cases (%)					
MASLD/MASH	19 (50.0)	N/A	N/A		
Overlapping MASLD/MASH and alcohol-associated ^†^	8 (21.1)	N/A	N/A		
Autoimmune liver disease	1 (2.6)	N/A	N/A		
Cryptogenic	10 (26.3)	N/A	N/A		
Creatinine, mg/dL (median, IQR)	0.90 (0.75–1.1)	0.90 (0.80–1.10)	0.90 (0.70–1.30)	>0.9999	>0.9999
Total bilirubin, mg/dL (median, IQR)	0.70 (0.45–1.20)	1.00 (0.70–1.70)	0.90 (0.60–1.50)	0.0486 *	0.1848
INR, ratio (median, IQR)	1.00 (1.00–1.10)	1.00 (1.00–1.10)	1.00 (1.00–1.10)	>0.9999	>0.9999
AFP, ng/mL (median, IQR)	30.00 (0.00–110.00)	20.00 (0.00–325.00)	10.00 (0.00–190.00)	>0.9999	>0.9999
ALT, mg/dL (median, IQR)	30.00 (17.00–48.50)	35.5 (21.00–61.00)	33.00 (20.00–71.25)	0.2937	0.5179
Platelet, 10^3^/μL (median, IQR)	201.5 (147.3–247.8)	181.0 (127.9–249.8)	145.5 (102.5–219.8)	>0.9999	0.0192 *
Fib-4 index (median, IQR)	2.230 (1.375–3.868)	2.855 (1.778–5.028)	3.965 (2.210–7.935)	0.2351	0.0012 **
MELD-Na score (median, IQR)	10.80 (6.90–13.74)	9.65 (7.47–14.34)	9.49 (7.47–14.47)	>0.9999	>0.9999
Child–Pugh score, cases (A/B/C)	20/8/1 ^‡^	226/61/26 ^‡^	62/22/7 ^‡^	0.4285	0.7051
HCC stage, cases (I/II/III/IV)	14/6/8/8 ^‡^	138/40/131/56 ^‡^	44/28/30/13 ^‡^	0.2983	0.3569

^†^ The alcohol-associated etiology includes patients with overlapping MASLD/MASH metabolic risks. ^‡^ Missing data. * and ** for *p* < 0.05 and <0.01. NBNC: Non-HBV and Non-HCV; HBV: Hepatitis B virus; HCV: Hepatitis C virus; BMI: Body mass index; MASLD/MASH: Metabolic dysfunction-associated steatotic liver disease/metabolic dysfunction-associated steatohepatitis; N/A: Not applicable; INR: International normalized ratio; AFP: Alpha-fetoprotein; ALT: Alanine transaminase; MELD: Model for End-stage Liver Disease; HCC: hepatocellular carcinoma.

**Table 2:** Comparison of characteristics of HBV, HCV and w/o etiology groups by gender, age, METAVIR score, and Edmondson score.

Characteristic	HBV, *n* = 10 ^1^	HCV, *n* = 9 ^1^	w/o ETIOLOGY, *n* = 15 ^1^	*p*-Value ^2^
Gender, cases				>0.9
Female	3 (30%)	3 (33%)	6 (40%)	
Male	7 (70%)	6 (67%)	9 (60%)	
Age	42 (28, 57)	66 (63, 68)	65 (54, 72)	0.005
METAVIR score, cases				<0.001
F0–F1	1 (10%)	1 (11%)	14 (93%)	
F2–F3	3 (30%)	1 (11%)	0 (0%)	
F4	6 (60%)	7 (78%)	1 (6.7%)	
Edmonson score, cases				>0.9
I–II	6 (60%)	5 (56%)	9 (60%)	
III–IV	4 (40%)	4 (44%)	6 (40%)	

^1^ *n* (%); Median (IQR), ^2^ Fisher’s exact test; Kruskal–Wallis rank sum test, HBV: Hepatitis B virus; HCV: Hepatitis C virus; w/o: Without.

## Data Availability

The datasets used and/or analyzed during the current study are available from the corresponding author on request.

## Abbreviations

The following abbreviations are used in this manuscript:

AFP	Alpha-fetoprotein
ALB	Albumin
ALD	Alcohol-associated liver disease
ALT	Alanine aminotransferase
AST	Aspartate aminotransferase
BMI	Body mass index
CD	Cluster of differentiation
CLP	Common lymphoid progenitors
CMP	Common myeloid progenitors
CT	Computed tomography
DC	Dendritic cells
DEG	Differentially expressed gene
EPC	Epithelial progenitor cell
FDR	False discovery rate
FIB-4	Fibrosis-4
GEO	Gene Expression Omnibus
GMP	Granulocyte myeloid progenitors
GSEA	Gene Set Enrichment Analysis
HBV	Hepatitis B virus
HCC	Hepatocellular carcinoma
HCV	Hepatitis C virus
HSC	Hematopoietic stem cell/Hepatic stellate cell
IHC	Immunohistochemistry
INR	International normalized ratio
IQR	Interquartile range
KRT7	Keratin 7
MAIT cell	Mucosal-associated invariant T cell
MASH	Metabolic dysfunction-associated steatohepatitis
MASLD	Metabolic dysfunction-associated steatotic liver disease
MELD-Na	Model for End-Stage Liver Disease (with Sodium)
MEP	Megakaryocyte–erythroid progenitor cells
MRI	Magnetic Resonance Imaging
MPP	Multipotent progenitors
MSC	Mesenchymal stem cells
NBNC	Non-HBV and non-HCV
NES	Normalized enrichment score
NK cells	Natural killer cells
qPCR	Quantitative PCR
Tgd cell	Gamma delta T cell
TNM	Tumor, node, metastasis
Treg	Regulatory T cell
